# Potential of UVC germicidal irradiation in suppressing crown rot disease, retaining postharvest quality and antioxidant capacity of *Musa* AAA “Berangan” during fruit ripening

**DOI:** 10.1002/fsn3.482

**Published:** 2017-05-17

**Authors:** Nuratika Tamimi S. Mohamed, Phebe Ding, Jugah Kadir, Hasanah M. Ghazali

**Affiliations:** ^1^ Department of Crop Science Faculty of Agriculture Universiti Putra Malaysia Serdang Selangor Malaysia; ^2^ Department of Plant Protection Faculty of Agriculture Universiti Putra Malaysia Serdang Selangor Malaysia; ^3^ Department of Food Science Faculty of Food Science and Technology Universiti Putra Malaysia Serdang Selangor Malaysia

**Keywords:** Antioxidant, cell wall, crown rot, peel browning, physico‐chemical quality, UVC

## Abstract

Crown rot caused by fungal pathogen is the most prevalent postharvest disease in banana fruit that results significant economic losses during transportation, storage, and ripening period. Antifungal effects of ultraviolet C (UVC) irradiation at doses varied from 0.01 to 0.30 kJ m^−2^ were investigated in controlling postharvest crown rot disease, maintenance of fruit quality, and the effects on antioxidant capacity of Berangan banana fruit during ripening days at 25 ± 2°C and 85% RH. Fruits irradiated with 0.30 kJ m^−2^ exhibited the highest (i.e., 62.51%) reduction in disease severity. However, the application of UVC at all doses caused significant browning damages on fruit peel except the dose of 0.01 kJ m^−2^. This dose synergistically reduced 46.25% development of postharvest crown and did not give adverse effects on respiration rate, ethylene production, weight loss, firmness, color changes, soluble solids concentration, titratable acidity, and pH in banana as compared to the other treatments and control. Meanwhile, the dose also enhanced a significant higher level of total phenolic content, FRAP, and DPPH values than in control fruits indicating the beneficial impact of UVC in fruit nutritional quality. The results of scanning electron micrographs confirmed that UVC irradiation retarded the losses of wall compartments, thereby maintained the cell wall integrity in the crown tissue of banana fruit. The results suggest that using 0.01 kJ m^−2^ UVC irradiation dose as postharvest physical treatment, the crown rot disease has potential to be controlled effectively together with maintaining quality and antioxidant of banana fruit.

## 1 Introduction

Banana fruit is one of the most economically important commodities recognized worldwide for its deliciousness, multiple uses, and health benefits to human. Banana fruit prized the developing countries and considered as one of the Malaysian premium fruit that aimed to be developed under Entry Point Project of National Key Economic Area for fruit production (ETP Annual Report, 2014). *Musa* AAA “Berangan” banana is a local dessert cultivar and popular among Malaysians and ASEAN countries (Ding & Darduri, 2009). During marketing, the fruits are commonly attacked with crown rot disease and ultimately caused major economic losses (Khan, Aked, & Magan, 2001). The rot symptoms occurred through blackening and softening at crescent shaped of crown tissue and thereafter spread into the pedicels of fingers in severe stages. The infected fruit usually causes abnormal ripening and finger drops where fruits are difficult to handle. The crown rot disease is particularly important during rainy season and the occurrence depends on transportation, packaging, and physiology of the fruits (Sangeetha, Usharani, & Muthukumar, 2010).

Until today, the application of systemic fungicide such as prochloraz and thiabendazole is still the primary practice for commercially control of the pathogenic fungi involved in banana crown rot since it has been introduced in the late 1960s (Lassois & de Lapeyre de Bellaire, 2014). However, due to the banning of several fungicides, researches for nonchemical control strategy against postharvest disease of fruits and vegetables have been intensified (Civello, Vicente, & Martinez, 2006). Although the disease control was easily achieved, the application of synthetic fungicides resulted carcinogenic effect to human health and also has potential of hazard to the environment. Moreover, a prolonged use of fungicide could reduce its effectiveness due to resistance build‐up of pathogen population (Anthony, Abeywickrama, Dayananda, Wilsonwijeratnam, & Arambewela, 2004). Therefore, several attempts have been made toward crown rot control through cultural, physical, and biological methods as alternatives to the synthetic fungicides (Lassois, de Lapeyre de Bellaire, & Jijakli, 2008). A considerable reduction of crown rot was achieved by treatment with antagonistic yeast (Bastiaanse, de Lapeyre de Bellaire, Lassois, Misson, & Jijakli, 2010), calcium chloride (Alvindia & Natsuaki, 2007), essential oils and their major components (Herath & Abeywickrama, 2008), antagonistic bacteria (Gunasinghe & Karunaratne, 2009), cinnamon extract, chitosan coating, and hot water treatment (Win, Jitareerat, Kanlayanarat, & Sangchote, 2007).

Another high potential physical control agent irradiation has been proposed by the use of ultraviolet C (UVC) to suppress several postharvest diseases on harvested produces. UVC is a nonionizing and nonthermal method which utilized ultraviolet light at wavelength of 190–280 nm. UVC is able to enhance the storability of fresh produces by triggering natural disease resistances (Chang‐hong, Lu‐yun, Xian‐ying, Xiao‐xu, & Tie‐jin, 2012). Several findings revealed the ability of UVC radiation in reducing deterioration of fruits and vegetables when applied after harvest, for instance, in the case of oranges (Canale, Benato, Cia, Haddad, & Pascholati, 2011; Gunduz, Juneja, & Pazir, 2015), peppers (Rodoni, Zaro, Hasperue, Concellon, & Vicente, 2015), “Tatsoi” baby leaves (Tomas‐Callejas, Oton, Artes, & Artes‐Hernandez, 2012), and button mushrooms (Guan, Fan, & Yan, 2012).

However, until recently, the effect of UVC irradiation on postharvest control of crown rot disease on Berangan banana fruit has not been studied yet. Therefore, more information is required to understand the impacts of UVC on crown rot disease control, ultrastructural modifications, physico‐chemical, and antioxidant changes occurred in the UVC‐irradiated fruits.

## 2 Materials and methods

### 2.1 Fruit material

Mature green stage of *Musa* AAA Berangan banana (second and third hands) fruits were obtained from Seri Kembangan market, Selangor. The banana hands were selected according to size and shape uniformity free from physical and microbial damages. The fruit hands were transported to the laboratory on the same day, washed with tap water, and surface disinfected by submerging in 0.05% (v/v) sodium hypochlorite solution. Then, after 2 min at ambient temperature, the hands were rinsed with two changes of sterilized distilled water to remove traces of bleaching agent followed by air drying at room temperature (25 ± 2°C/85% RH). The banana hands were then separated into clusters using a sterilized curved knife. Each cluster consisted of four fingers.

### 2.2 UVC irradiation treatment

A stainless steel chamber equipped with UVC irradiation facility provided by Vision Scientific Co. Ltd. (Malaysia) was used in this study. The chamber contained a low pressure mercury vapor discharge lamp (90 cm long, 220 V, and 36 W) and emitted 254 nm of UVC light (VER Bright, Vision Scientific, Korea). The interior of chamber was lined with reflective mirrors which designed to minimize any shadowing effect on irregularly shaped samples. Before application, UVC light was switched on for 30 min to stabilize the emission of radiation.

Initially, the UVC dose rate was obtained using a digital radiometer (UVC‐254, Lutron Electronic Enterprise, Taiwan) and calibrated to read specifically at 254 nm before being used. The dose rate at eight areas inside the chamber was measured and averaged with a value of 0.336 W m^−2^. Thereafter, various UVC doses used in this study were determined based on differential irradiation timing under the obtained fixed dose rate of 0.336 W m^−2^ according to Stevens et al. (1998), and expressed in kJ m^−2^. The calculation used was as follows: UVC dose (kJ m^−2^) = Dose rate (Wm^−2^)  × Irradiation time (s) × 10^−3^.

Banana clusters (four fingers per cluster) each were exposed to UVC light at different irradiation times, that is, 0, 30, 60, 90, 120, 180, 300, 600, and 900 s which equivalent to 0, 0.01, 0.02, 0.03, 0.04, 0.06, 0.10, 0.20, and 0.30 kJ m^−2^ of UVC doses, respectively. Nonirradiated fruits were used as negative control while banana that was dipped with 1.10 g L^−1^ Octave^®^ fungicide solution (active ingredient: 50% w/w prochloraz) for 2 min was used as positive control treatment. Each treatment comprised of three banana clusters and a total of 30 clusters were used.

### 2.3 Ripening process

Immediately after UVC treatment, banana fruits were initiated for uniform ripening using 10 mL L^−1^ of ethylene in a sealed airtight polyethylene bag and left for 24 hr at room temperature (25 ± 2°C). After 24 hr, the ethylene gas was released and the fruits were allowed to ripen at the same condition. Analysis was performed on day 0 (before ripening initiation) and day 1, 3, and 5 (after ripening initiation).

### 2.4 Crown rot disease assessments

Crown rot severity was assessed using a disease severity scale of 0–7 by Alvindia, Kobayashi, Natsuaki, and Tanda (2004) with modifications, where 0 = 0% of crown discoloration and mycelial growth, 1 = 1–20% crown discoloration or mycelial growth and limited to the surface of cut crown, 2 = 21–40% crown discoloration or mycelial growth on crown area, 3 = 41–60% crown discoloration or mycelial growth on crown area, 4 = 61–80% crown discoloration or mycelial growth on crown area, 5 = 81–100% crown discoloration or mycelial growth on crown area, 6 = 100% with crown discoloration or mycelial growth progressing toward the pedicels, and 7 = 100% with rot on fruit pedicels, peels, and fingers were easily to drop‐off when handled. Disease severity was expressed according to the formula given by Campbell and Madden (1990):

$$\begin{array}{cc} \text{DS}\;\;(\%)= & \frac{\sum (\text{Severity rating}\times \text{number of fruit clusters in that rating)}}{\text{Total number of fruit clusters assessed}\times \text{highest scale}}\\ & \times 100\% \end{array}$$

Area under disease progress curve (AUDPC) was calculated according to the formula given by Capdeville, Wilson, Beer, and Aist (2002):

$$\text{AUDPC}=\sum [\left(y_{i}+y_{i+1}\right)/2\times \left(t_{i+1}-t_{i}\right)]$$

where, y_i_ = Disease severity at time t_i_; y_i+1 _= Disease severity at time t_1+1_; t = Time in days

### 2.5 Browning assessment

A subject hedonic score of browning discoloration was evaluated based on the appearance of total brown area on banana fruit surface. The scales used were as follows: 1 =  no browning, 2 = <20% of the peel surface, 3 = 20–40% of the peel surface, 4 = 40–60% of the peel surface, and 5 = >60% of the peel surface (Ding & Ling, 2014).

### 2.6 Banana crown tissue under scanning electron microscope

The effects of UVC irradiation on surface and cell wall modifications in crown tissue of Berangan banana were examined using a scanning electron microscope (SEM). Initially, sample blocks measuring 1 cm^3^ were excised from crown tissue of banana hand and then fixed in 4% glutaraldehyde for 48 hr in vacuum condition (0.05 MPa). After fixation, samples were washed three times with 0.1 mol/L sodium cacodylate buffer at pH 7.2 and postfixed in 1% (w/v) osmium tetroxide for 2 hr at 4°C. The samples were washed three times with 0.1 mol/L sodium cacodylate for 30 min each. A series of dehydration was performed in five acetone concentrations (35, 50, 75, 95, 100%) for 30 min each. After that, samples were critical‐point‐dried using a Balzer CPD 030 (Balzer Union, Furstentum, Liechtenstein) and the dried samples were stuck on aluminium stubs followed by gold coating in a Polaron sputter coater. Finally, the samples were viewed under SEM (JOEL JSM 6400, Ltd., Japan) at acceleration voltage of 15 kV and working distance of 15 mm.

### 2.7 Determination of respiration rate and ethylene production

Respiration rate and ethylene production of banana fruit was determined by incubating individual finger of banana in a 1.9 L stackable airtight container for 2 hr at room temperature (25 ± 2°C) after measuring its weight and volume. After incubation, 1 ml of gas aliquot was withdrawn from the headspace using a gas‐tight syringe for analysis of carbon dioxide and ethylene production. The gas aliquot was injected into a gas chromatograph (Clarus 500, Perkin Elmer, Shelton) equipped with stainless steel column (3 m × 3.125 mm; Porapak Q 50/80 mesh) (Supelco, Sigma‐Aldrich, St Louis, MO) with a flame ionization (150°C) and thermal conductivity (150°C) detectors to detect ethylene and carbon dioxide, respectively. Nitrogen at a flow rate of 45 ml min^−1^ was used as a carrier gas. The amount of carbon dioxide was expressed in mL CO_2_ kg^−1 ^hr^−1^ while ethylene production was expressed in μl C_2_H_4_ kg^−1 ^hr^−1^.

### 2.8 Determination of weight loss

Fruit weight loss was determined by the differences in fruit weights at day 0, 1, 3, and 5 which were compared with day 0 and expressed in percentage. Three fruit fingers in each replicates per treatment were marked and used for weighing at particular day interval using an electronic balance (EK‐600H, Japan).

### 2.9 Determination of peel color

Banana peel color was measured using a chroma meter (CR‐400, Minolta Corp., Japan) at three regions of fruit surface, that is, stem end, equatorial region, and floral end. The readings were expressed in L*, C*, and h° values, indicating the fruit lightness, chroma, and hue angle, respectively.

### 2.10 Determination of fruit firmness

Peel and pulp firmness measurements were performed using an Instron Universal Testing Machine (Model 5540 load frame, Instron Corp.,) with compression mode. Maximum force required for compressing by 10 mm depth into the peel and pulp was expressed in Newton (N). Peel firmness was taken in three regions on the fruit surface, that is, stem end, equatorial region, and floral end. The finger was then sliced horizontally into 1‐cm‐thick of the same regions described above to measure the pulp firmness. Three readings were recorded from three fruit fingers in each of the four replicates per treatment and the mean of force values (N) was calculated.

### 2.11 Determination of soluble solids concentration

Soluble solids concentration in fruit pulp was determined using a digital refractometer (Model N‐1 α Atago, Japan). Ten gram of banana pulp tissue was homogenized with 90 mL distilled water using a kitchen blender (MX‐900M, Panasonic, Malaysia) and filtered through a funnel with cotton wool. Two drops of filtrate was placed on a clean prism of the refractometer using a plastic dropper. The readings were multiplied by dilution factor (10) to obtain %SSC of the pulp tissue.

### 2.12 Determination of titratable acidity and pH

The remainder of filtered juice from SSC determination was used to measure titratable acidity and pH of banana pulp (Ranggana, 1977). Five millimeter of the filtrate was transferred into a 100 ml conical flask and added with three drops of 1% (v/v) phenolphthalein as indicator. The sample was titrated with 0.1 mol/L sodium hydroxide (NaOH) solution until the color changed to light pink and persistent at least 15 s. The titer volume in ml was recorded and the result was expressed as percentage of anhydrous malic acid. Meanwhile, the pH of banana pulp was measured using a glass electrode pH meter (Crison Instruments, S.A., Barcelona).

### 2.13 Determination of total phenolic content

The extraction of banana pulp tissue for total phenolic and antioxidant assays followed the method described by Shian, Abdullah, and Musa (2012). Twenty gram of banana pulp were frozen immediately in liquid N_2_ and ground using a dry kitchen chopper for 10 s and then kept at −20°C. Five gram of frozen pulp tissue was dissolved in 70% (v/v) acetone. After that, the homogenate was extracted in darkness by swirling on orbital shaker at 180 rpm for 1 hr and then centrifuged at 3,000 g for 10 min before decanting the supernatants for total phenolic content and antioxidant analyses.

Total phenolic content was determined following the method described by Singleton and Rossi (1965). An aliquot of 300 μl supernatant extract and 1.5 ml of 10% (v/v) Folin‐Ciocalteu reagent were mixed in test tube and incubated for 5 min in darkness, followed by addition with 1.2 ml of 6% (w/v) of sodium carbonate. The mixture was incubated in darkness for 1 hr at room temperature before measuring the absorbance at 765 nm using a spectrophotometer (S1200, Spectrowave spectrophotometer, Cambridge, England). Total phenolic content was expressed in mg gallic acid equivalents (GAE) g^−1^ fresh weight.

### 2.14 Determination of antioxidant capacity

The antioxidant capacity of banana pulp was measured using ferric reducing antioxidant power (FRAP) (Benzie & Strain, 1996) and 1,1‐diphenyl‐2‐picrylhydrazyl (DPPH) radical scavenging activity (Brand‐Williams, Cuvelier, & Berset, 1995) assays. In FRAP assay, the FRAP reagent was freshly prepared by mixing 10 mmol/L of 2,4,6‐tris(2‐pyridyl)‐s‐triazine (TPTZ), 0.3 mol/L acetate buffer (pH 3.6), and 20 mmol/L ferric chloride in the ratio of 1:10:1 (v/v/v). An aliquot of 40 μl of sample extracts were added to 3 ml of FRAP reagent and incubated in a water bath at 37°C for 1 hr. Absorbance was measured at 593 nm against a control that was prepared by adding 40 μl of 70% acetone to 3 ml of FRAP reagent. The obtained results were expressed as μmol trolox equivalent (TE) g^−1^ of banana fresh weight using standard linear regression curve.

In DPPH radical scavenging activity assay, 0.1 ml of sample extracts were added to 3.9 ml of 1 mmol/L DPPH solution (prepared in acetone), vortex‐mixed, and then kept in the dark for 30 min. The absorbance was measured at 517 nm. Meanwhile, 0.1 ml of 70% acetone was added to 3.9 ml of DPPH reagent as the control solution. The results were expressed as percentage inhibition of DPPH and calculated using the following equation:

$$\%\;\;\text{DPPH inhibition}=\frac{\left(A_{0}-A_{1}\right)}{A_{0}}\times 100\%$$

where, A_0_ = Absorbance of the control, A_1 = _Absorbance of sample

### 2.15 Statistical analysis

The experiment was conducted in a completely randomized design with factorial arrangement of treatment (10 levels of UVC doses × 5 ripening days) with three replications. Each treatment comprised of three banana clusters and a total of 30 clusters were used. The experiment was repeated twice. Data were analyzed using analysis of variance and means were compared using Duncan's multiple range test (DMRT) at a significance level of *p* ≤ .05 using SAS statistical analysis software version 9.3 (SAS Institute Inc., Cary, North Carolina). Data in percentages were transformed using arcsine transformation before determining the significance level using DMRT.

## 3 Results

### 3.1 Effect of UVC irradiation in suppressing crown rot disease severity

**Figure 1:**
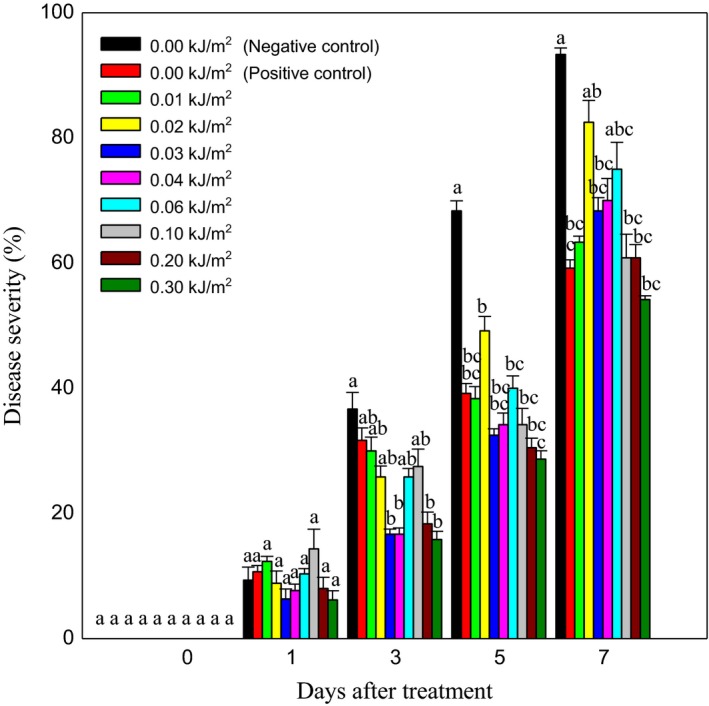
Effect of different ultraviolet C doses on crown rot disease severity that naturally infected on Berangan banana fruit during ripening at 25 ± 2°C and 85% RH. The different letters in each day are significantly different by Duncan's multiple range tests at *p* ≤ .05 on arcsine‐transformed values. Data are means of four replicates, each of three banana clusters per treatment. Vertical bars indicate SE of means

The effect of UVC irradiation at various doses on crown rot disease severity (DS) of Berangan banana fruit during days after ripening initiation is shown in Figure 1. UVC‐treated and positive control fruits did not show any significant differences in reduction of crown rot severity at the beginning of ripening. By 3 days after UVC treatment, the severity increased with significant higher percentage of DS in negative control fruits than the rest of fruits especially at day 5. By day 7, a similar trend of DS as day 5 was found in the fruit. The results obtained generally indicated that the efficacies of UVC irradiation in controlling Berangan banana fruit crown rot severity were comparable with fungicide application.

Area under disease progress curve (AUDPC) was also calculated to evaluate the efficacy of UVC treatment against disease progress over time (Table 1). At the end of ripening days, the highest AUDPC of 77.5 unit^2^ was recorded in negative control fruits while fruits treated with 0.30 kJ m^−2^ showed the lowest AUDPC value of 38.1 unit^2^.

**Table 1:** Effect of different UVC doses on area under disease progress curve (AUDPC) and reduction of crown rot disease of Berangan banana fruit ripened at 25 ± 2°C and 85% RH

UVC doses (kJ m^−2^)	AUDPC (unit^2^)	Disease reduction over control (%)
Positive control	54.21 bc^a^	46.66 ab
Negative control	77.50 a	Not applicable^b^
0.01	54.63 bc	46.25 ab
0.02	63.52 ab	37.49 b
0.03	44.04 bc	56.66 ab
0.04	45.79 bc	54.94 ab
0.05	55.54 abc	45.35 ab
0.10	51.42 bc	49.41 ab
0.20	42.63 bc	58.06 ab
0.30	38.10 c	62.51 a

a Mean values in each column followed by the same letter are not significantly different at *p* ≤ .05 ^b^according to Duncan's multiple range test.

The efficacy of UVC irradiation in reducing the crown rot severity rate on Berangan banana fruit was expressed in percentage disease reduction (Table 1). Results showed that there were no significant differences in percentage of disease reduction between all UVC‐treated and positive control fruits. This indicated that UVC gave the similar effect as fungicide in reducing the disease, ranged from 37.49% to 62.51% compared to nonirradiated fruits.

Crown tissue softening and discoloration are the important symptoms that corresponded to the severity of crown rot disease on banana fruit. In this experiment, the changes of crown tissue in terms of color and texture as influenced by UVC irradiation were evaluated (Table 2). Negative control and fruit treated with 0.04 kJ m^−2^ UVC dose attained high scores of crown discoloration, indicating that the crown showed rapid changes from green to yellow/green color. Meanwhile, the fruit treated with 0.20 kJ m^−2^ retained lowest score in crown color with green color.

**Table 2:** Effects of UVC irradiation doses on crown tissue discoloration and softening on Berangan banana ripened at 25 ± 2°C and 85% RH

UVC doses (kJ m^−2^)	Degree of severity on crown tissue	
	Crown discoloration score (1–7)^a^	Crown softening score (0–4)^c^
Positive control	2.60 d^b^	1.67 b
Negative control	3.20 a	2.03 a
0.01	2.60 d	0.97 e
0.02	2.80 cd	1.57 bcd
0.03	2.60 d	2.00 a
0.04	3.07 ab	1.30 d
0.05	2.73 cd	1.37 cd
0.10	2.27 e	1.33 cd
0.20	1.87 f	1.67 b
0.30	2.90 bc	2.03 a

a Crown discoloration scores: 1 = Green, 2 = Green/yellow, 3 = Yellow/green, 4 = Yellow, 5 = Yellow/black, 6 = Black/yellow, and 7 = Black.

b Means followed by the same letter in the same column are not significantly different at *p* ≤ .05 on the arcsine‐transformed values according to DMRT.

c Crown softening scores: 0 =  Hard, 1 = 1–25% of crown tissue softening, 2 = 26–50% of crown tissue softening, 3 = 51–75% of crown tissue softening, and 4 = 76–100% of crown tissue softening.

Negative control, 0.03 and 0.30 kJ m^−2^ UVC‐treated fruits exhibited significantly (*p* ≤ .05) higher degree of crown softening than the other treatments. In contrast, fruits treated with 0.01 kJ m^−2^ UVC showed the lowest score of crown softening, indicating the dose was able to retain the crown tissue texture. The tissue of crown appeared hardened by the UVC treatment and this indirectly delayed the rotting symptoms.

### 3.2 The optimum dose of UVC irradiation on Berangan banana fruit

The application of UVC irradiation dose on banana fruit was found limited due to the adverse effect of higher doses which caused browning discoloration on the fruit peel (unpublished data). Therefore, in this experiment, the lower doses of UVC (0.00–0.05 kJ m^−2^) were tested in order to determine the optimum dose of UVC irradiation on Berangan banana fruit.

**Figure 2:**
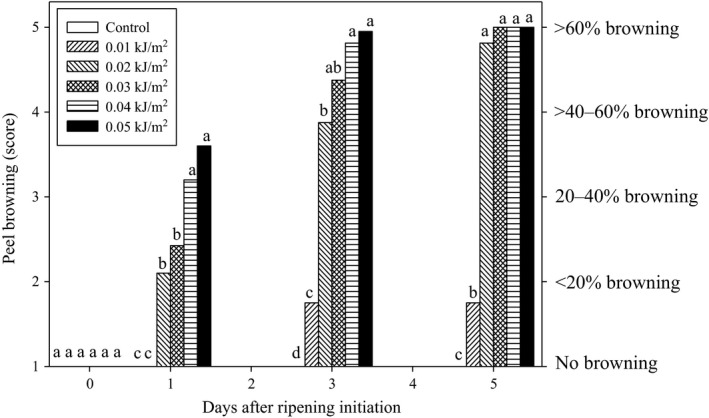
Effect of ultraviolet C irradiation doses on peel browning of Berangan banana ripened at 25 ± 2°C and 85% RH for 5 days. The different letters in each day are significantly different by Duncan's multiple range tests at *p* ≤ .05 on arcsine‐transformed values. Data are means of four replicates, each of three banana clusters per treatment

No visible damage was observed on UVC‐treated fruits immediately after treatment exposure at day 0 before the ripening initiation (Figure 2). After 24 hr of ripening initiation (I DAR), fruits treated with 0.04 and 0.05 kJ m^−2^ UVC doses showed significantly higher browning scores than fruits treated with 0.03 and 0.02 kJ m^−2^ UVC, although control and 0.01 kJ m^−2^ UVC‐treated fruits remained browning free at the same day. By 3–5 DAR, the browning on fruit treated with UVC doses of 0.02–0.05 kJ m^−2^ showed browning scores that corresponded to 40–60% peel browning. Fruits that treated with 0.01 kJ m^−2^ UVC showed a much lower browning score among all the UVC‐treated fruits by 5 DAR. This result suggested that the threshold for commencement of peel browning in Berangan banana fruit was 0.01 kJ m^−2^ UVC.

### 3.3 Changes of crown surface morphology after UVC irradiation

**Figure 3:**
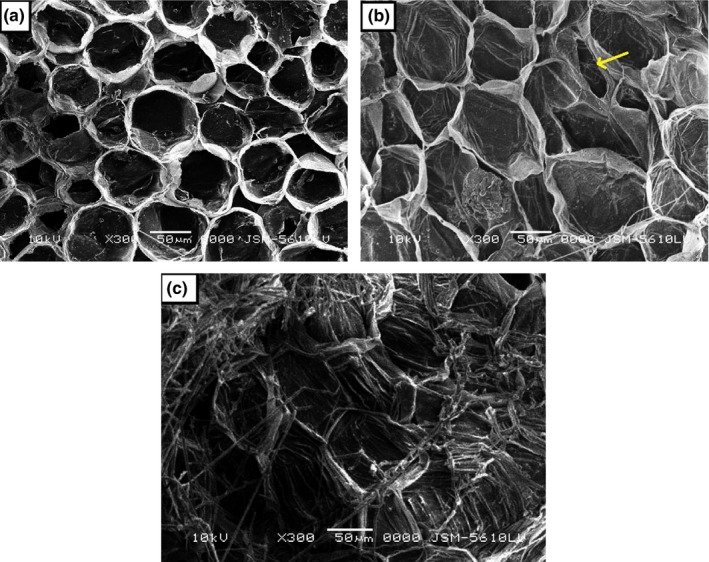
SEM micrographs of Berangan banana crown tissue structures on day 1 and 5 after ripening initiation. (a) Control crown tissue at day 1. Cells have an intact polyhedral interlocking profile with limited intercellular spaces. (b) 0.01 kJ m^−2^ ultraviolet C‐treated crown tissue at day 5. The cells of ultraviolet C‐irradiated crowns retained its cellular compartment, but with prominent intercellular spaces (arrow). (c) Control crown tissue at day 5. Cells showed an intensive loss of cellular structure due to development of fungal mycelia in the crown tissue. (Bars = 50 μm, x300)

The effect of UVC irradiation on modification of crown cell wall structure on Berangan banana fruit was viewed and illustrated in Figure 3. There were no obvious changes observed in the cell packing in UVC‐treated and control crowns at day 1 after ripening initiation. The cells have angular polyhedral profile with limited intercellular spaces (Figure 3a). By day 5, the crowns treated with UVC retained the cellular profile, but its intercellular spaces have increased (Figure 3b). In contrast, the interfaces between cells of control crown tissue became indistinct, suggesting intensive losses of cell compartment has occurred in (Figure 3c). The disappearance of cell packing might also relate to the necrotic lesion and severe tissue dehydration.

### 3.4 Effects of UVC irradiation on respiration rate and ethylene production

**Figure 4:**
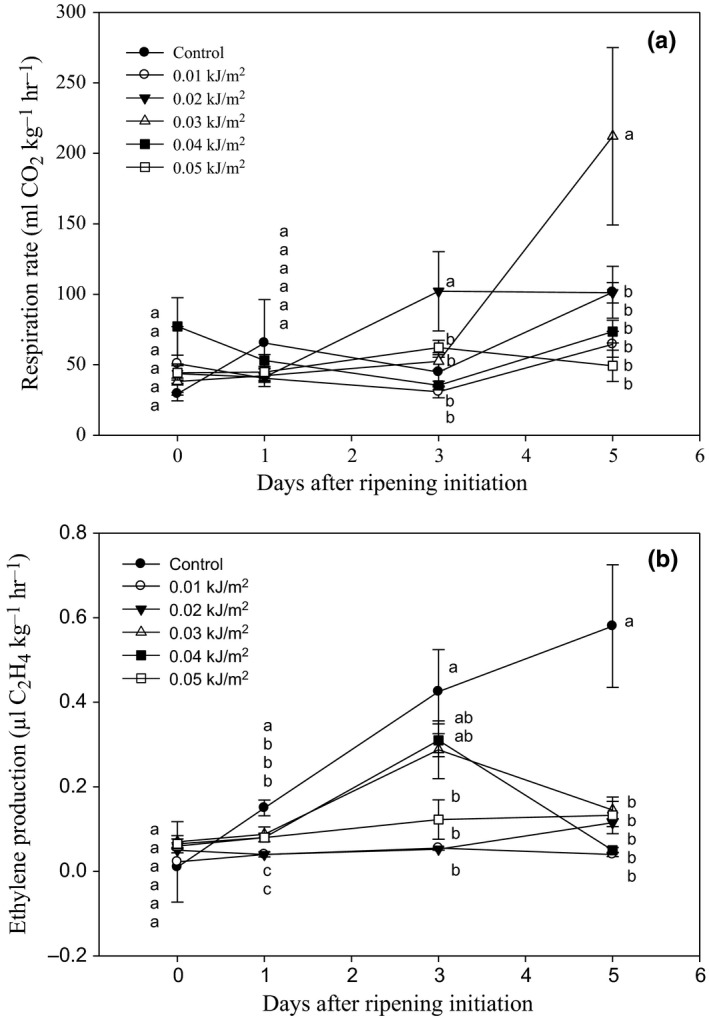
Changes in (a) respiration and (b) ethylene production rates of Berangan banana fruit as influenced by different ultraviolet C irradiation doses during ripening days at 25 ± 2°C and 85% RH. The different letters in each day are significantly different at *p* ≤ .05 according to Duncan's multiple range test. Data are means of four replicates, each of three banana fingers per treatment. Vertical bars indicate S.E. of means.

The initial respiration rate of all fruits ranged from 29.39 to 76.72 mL kg^−1 ^hr^−1^ (Figure 4a). By 5 DAR, no significant differences were observed between respiration rate among all UVC‐treated fruits and the controls, except for fruit treated with 0.03 kJ m^−2^ UVC which exhibited significant higher concentration of CO_2_ than others.

The initial ethylene concentrations were 0.01 μL kg^−1 ^hr^−1^ and 0.02–0.07 μL kg^−1 ^hr^−1^ for control and UVC‐treated fruits, respectively (Figure 4b). The ethylene production in control fruit increased throughout ripening day, reaching the highest concentration of C_2_H_4_ production by day 5, as compared to all UVC‐treated fruits. This result indicated that the suppressive effect of UVC irradiation on ethylene production on banana fruit during ripening.

### 3.5 Effects of UVC irradiation on physico‐chemical characteristics

#### 3.5.1 Weight loss

UVC irradiation did not affect weight loss of fruit (Table 3). However, there was a significant difference in percentage weight loss as days after ripening initiation progressed. The total weight loss of banana fruit ripened from day 0 to 5 was observed with the value of 8.62%. There was a sharp increase of weight loss when the fruit ripened from day 1 to 3 (338%), followed by a 51% weight loss as fruit ripened from day 3 to 5.

**Table 3:** Main and interaction effects of UVC irradiation doses and days after ripening initiation on percentage weight loss of Berangan banana fruit ripened at 25 ± 2°C and 85% RH for 5 days

Factors	Weight loss (%)
UVC doses, kJ m^−2^ (U)	
Control	3.84 a^b^
0.01	3.88 a
0.02	4.06 a
0.03	3.92 a
0.04	4.06 a
0.05	4.16 a
Days after ripening initiation (DAR)	
0	a
1	1.30 c
3	5.69 b
5	8.62 a
Interaction	
U × DAR	n.s

n.s, Nonsignificant different at *p* ≥ .05.

a Not applicable.

b Means followed by the same letter in the same column within factors are not significantly different at *p* ≤ .05 according to DMRT.UVC, ultraviolet C

#### 3.5.2 Peel color

**Figure 5:**
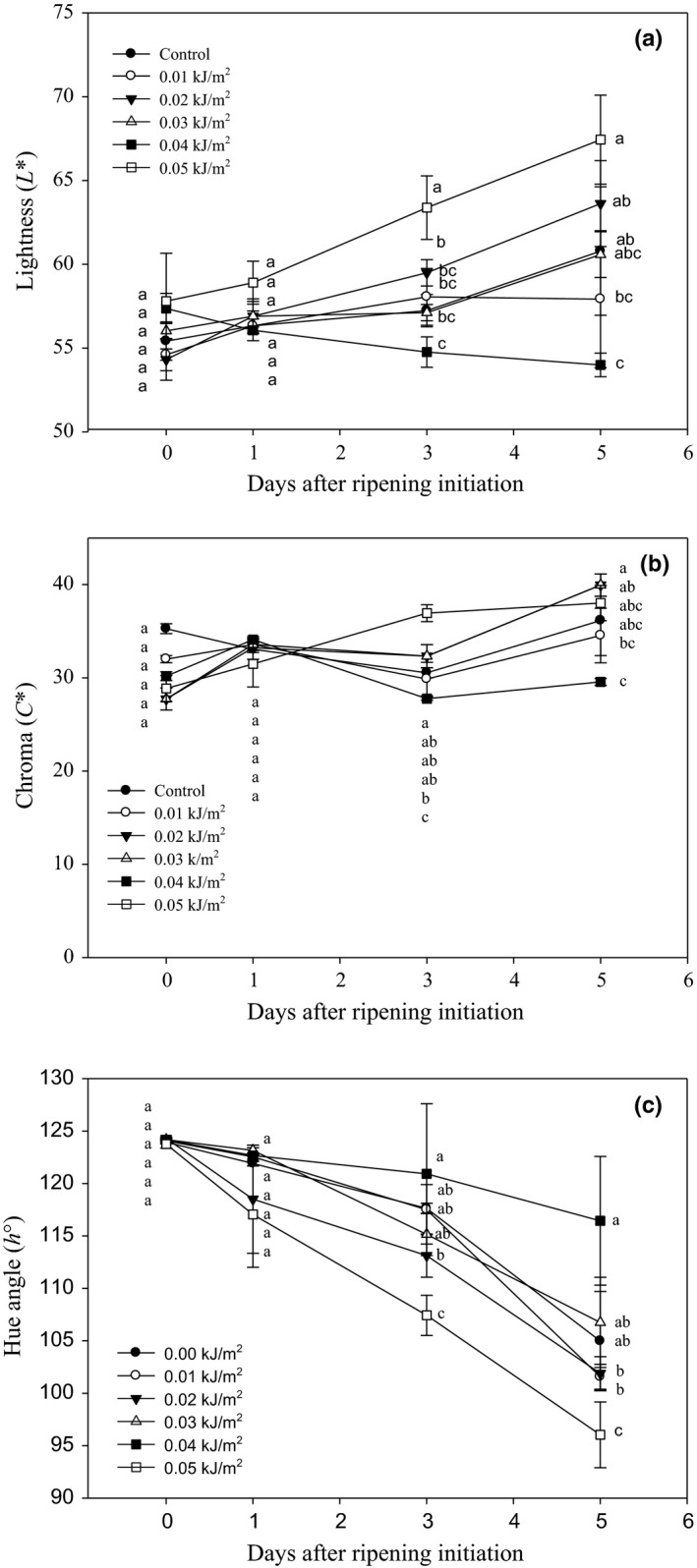
Changes in peel (a) lightness (L*), (b) chroma (C*), and (c) hue angle (h°) of Berangan banana fruit as influenced by different ultraviolet C irradiation doses during ripening days at 25 ± 2°C and 85% RH. The different letters in each day are significantly different at *p* ≤ .05 according to Duncan's multiple range test. Data are means of four replicates, each of three banana fingers per treatment. Vertical bars indicate SE of means.

The changes in banana peel color occurred progressively from green to yellow when the fruit started to ripen, as indicated by the increasing trend in fruit lightness (54.31–67.44) (Figure 5a), chromaticity (27.85 to 39.95) (Figure 5b), and the decreasing trend in hue angle (124.44–96.04) (Figure 5c). Application of 0.05 kJ m^−2^ UVC significantly increased the peel lightness compared to the other treatments. In peel chromaticity, the fruits treated with 0.05 kJ m^−2^ showed a much higher C* values (36.94) as compared to control fruits (30.54) by 3 DAR (Figure 5b). Regardless of treatment, the hue angle of banana fruits decreased as DAR progressed. By day 5 after ripening initiation, fruit irradiated with 0.05 kJ m^−2^ showed the lowest h° which indicated that this fruit has more yellow than others (Figure 5c). Generally, these results showed that UVC at dose of 0.05 kJ m^−2^ enhanced rapid color changes in Berangan banana fruit.

#### 3.5.3 Firmness

**Figure 6:**
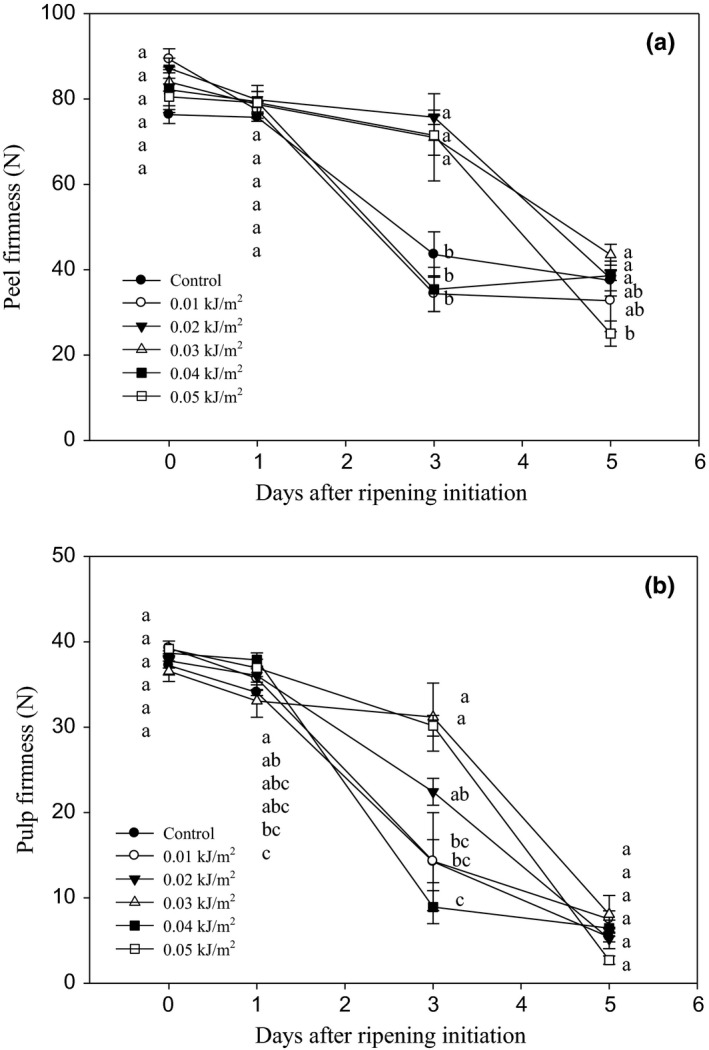
Changes in (a) peel and (b) pulp firmness of Berangan banana fruit as influenced by different ultraviolet C irradiation doses during ripening days at 25 ± 2°C and 85% RH. The different letters in each day are significantly different at *p* ≤ .05 according to Duncan's multiple range test. Data are means of four replicates, each of three banana fingers per treatment. Vertical bars indicate SE of means.

The initial firmness of banana peel was at the maximum, ranging between 76.97 and 89.32 N (Figure 6a). The values decreased progressively as DAR progressed. By 3 DAR, fruits treated with 0.02, 0.03, and 0.05 kJ m^−2^ UVC retained higher peel firmness than control, 0.01 and 0.04 kJ m^−2^ UVC‐treated fruits. However, there were no significant differences found in the peel firmness of control and UVC‐treated fruits by the end of ripening day.

Similar to peel, pulp firmness for all fruits decreased with the progress of DAR (Figure 6b). By 3 DAR, fruits treated with 0.03 and 0.05 kJ m^−2^ UVC doses exhibited a delay in pulp softening with a relatively higher value of firmness than control fruit (14.24 N). However, no significant differences were observed among all fruits by day 5. Generally, the results indicated that UVC irradiation did not retain the peel and pulp firmness of Berangan banana fruit at the end of ripening. The maintenance of fruit firmness was observed only until day 3 of ripening when fruit irradiated with 0.03 and 0.05 kJ m^−2^ UVC doses, but this effect was lost as ripening progressed.

#### 3.5.4 Soluble solids concentration, titratable acidity, and pH

**Figure 7:**
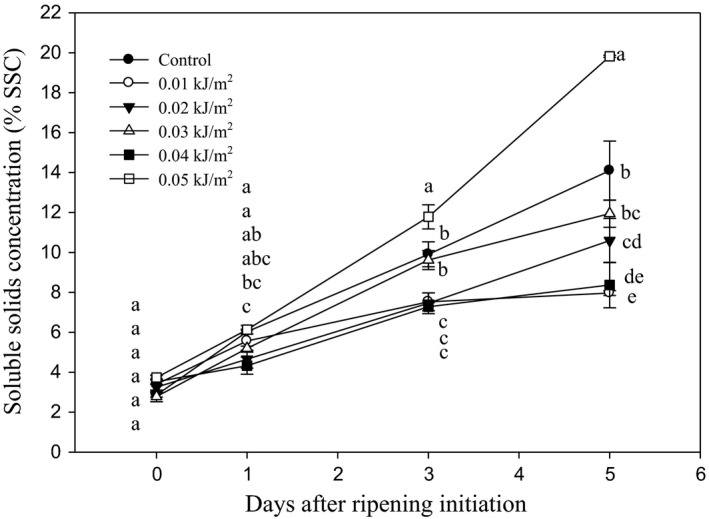
Changes in the soluble solids concentration of Berangan banana fruit as influenced by different ultraviolet C irradiation doses during ripening days at 25 ± 2°C and 85% RH. The different letters in each day are significantly different at *p* ≤ .05 according to Duncan's multiple range test. Data are means of four replicates, each of three banana fingers per treatment. Vertical bars indicate SE of means

The effect of UVC irradiation on SSC of banana fruit during ripening is shown in Figure 7. Generally, the SSC increased as DAR progressed. The initial SSC levels were 2.86 and 2.78–3.74% for control and UVC‐treated fruits, respectively. The values increased and reached the highest level by 5 DAR in UVC‐treated fruit especially those irradiated with 0.05 kJ m^−2^ UVC. The SSC of fruit irradiated with 0.01 and 0.04 kJ m^−2^ were the lowest by 5 DAR, suggesting that the doses were effective in delaying the increase of fruit SSC during ripening.

**Figure 8:**
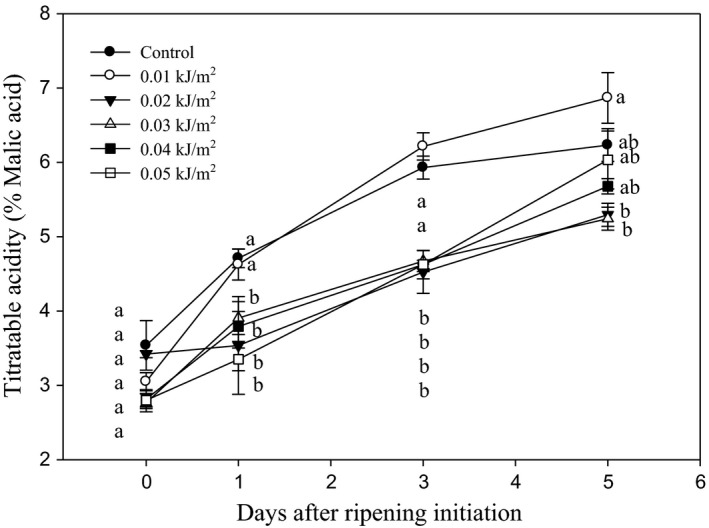
Changes in titratable acidity of Berangan banana fruit as influenced by different ultraviolet C irradiation doses during ripening days at 25 ± 2°C and 85% RH. The different letters in each day are significantly different at *p* ≤ .05 according to Duncan's multiple range test. Data are means of four replicates, each of three banana fingers per treatment. Vertical bars indicate SE of means.

Titratable acidity in control and UVC‐treated fruits increased as DAR progressed (Figure 8). The initial concentrations of titratable acidity were 3.54 and 2.28–3.42% for control and UVC‐treated samples, respectively. By 3 DAR, both control and 0.01 kJ m^−2^ UVC‐treated fruits showed significantly higher titratable acidity as compared to fruits treated with UVC at higher doses, that is, 0.02–0.05 kJ m^−2^. However, by day 5, there were no significant differences found in the titratable acidity among the fruits.

**Figure 9:**
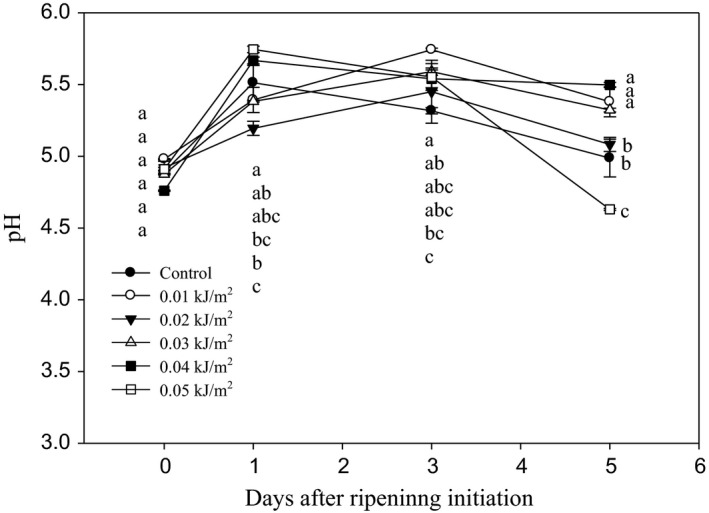
Changes in the pH of Berangan banana fruit as influenced by different ultraviolet C irradiation doses during ripening days at 25 ± 2°C and 85% RH. The different letters in each day are significantly different at *p* ≤ .05 according to Duncan's multiple range test. Data are means of four replicates each of three banana fingers per treatment. Vertical bars indicate SE of means

The changes of pH in banana fruit as influenced by different doses of UVC irradiation during DAR are shown in Figure 9. There were no significant differences observed in all fruit from day 0 until 3 DAR. By 5 DAR, the lowest pH of 4.63 was recorded in fruit treated with 0.05 kJ m^−2^ UVC, whereas, the higher pH ranging from 5.32 to 5.50 were found in fruits treated with UVC doses of 0.01, 0.03, and 0.04 kJ m^−2^.

### 3.6 Effects of UVC irradiation on total phenolic content and antioxidant capacity

**Figure 10:**
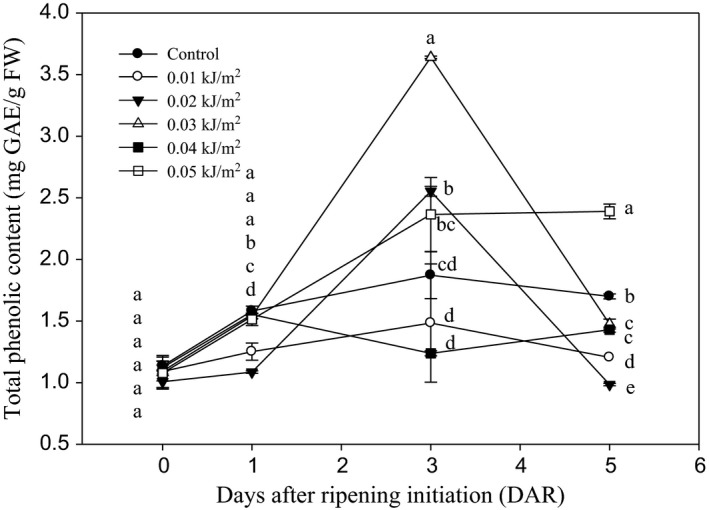
Changes in the total phenolic content of Berangan banana fruit as influenced by different ultraviolet C irradiation doses during ripening days at 25 ± 2°C and 85% RH. The different letters in each day are significantly different at *p* ≤ .05 according to Duncan's multiple range test. Data are means of four replicates, each of three banana fingers per treatment. Vertical bars indicate SE of means

The TPC of Berangan banana fruit increased as DAR progressed and by day 3; 0.03 kJ m^−2^ UVC‐irradiated fruit showed highest TPC among control and irradiated fruits (Figure 10). However, this fruit showed a sharp decreased by 5 DAR. The loss of TPC might be due to the higher rate of respiration that occurred in 0.03 kJ m^−2^ UVC‐irradiated fruits as indicated in previous experiment (Soluble solids concentration, titratable acidity, and pH 3.5.4). However, fruit treated with 0.05 kJ m^−2^ UVC exhibited highest amount of TPC by day 5 of ripening, indicating that the dose had induced more phenolic compounds in banana fruit.

**Figure 11:**
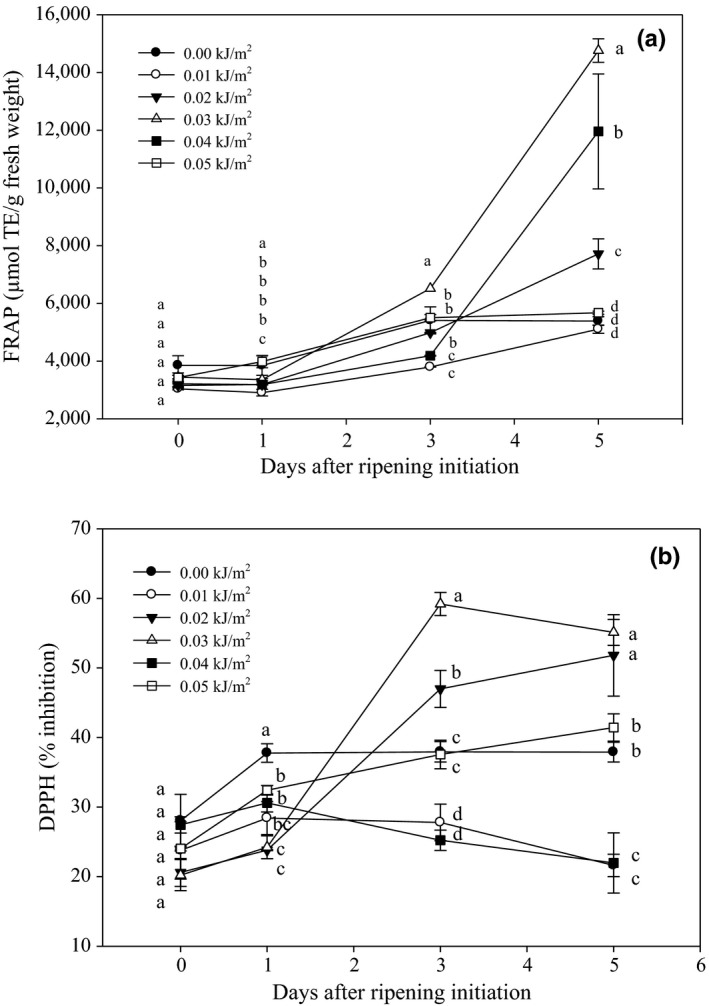
Changes in (a) ferric reducing antioxidant power and (b) DPPH activity of Berangan banana fruit as influenced by different ultraviolet C irradiation doses during ripening days at 25 ± 2°C and 85% RH. The different letters in each day are significantly different at *p* ≤ .05 according to Duncan's multiple range test. Data are means of four replicates each of three banana fingers per treatment. Vertical bars indicate SE of means

Meanwhile, FRAP activity of Berangan banana increased gradually throughout DAR (Figure 11a). By 3 DAR, 0.03 kJ m^−2^ UVC‐irradiated fruit exhibited higher antioxidant capacity among control and irradiated fruits. The trend extended until day 5, whereas control and the other UVC treatments showed a significant lower antioxidant capacity. The effect of UVC doses on antioxidant capacity in Berangan banana fruit as measured in DPPH radical scavenging activity during ripening is shown in Figure 11b. As ripening progressed to 3 DAR, the maximum scavenging activity of DPPH was observed in fruit treated with 0.03 kJ m^−2^ UVC followed by 0.02 kJ m^−2^, thereafter both treatments retained highest DPPH activity until the end of experiment.

## 4 Discussion

### 4.1 Effect of UVC irradiation in reducing crown rot disease on naturally infected fruits

In this study, UVC irradiation at 0.30 kJ m^−2^ dose reduced the crown rot disease infection on Berangan banana fruits as much as 62.51% compared to nonirradiated control fruits (Table 1). All UVC doses gave the similar effects as the application of postharvest fungicide, Prochloraz. Similar finding was reported in Valencia orange fruits, where UVC at doses of 7.28 and 15.66 kJ m^−2^ were able to control the incidence of citrus black spot disease that was naturally infected on fruits during storage period (Canale et al., 2011). Nigro, Ippolito, Lattanzio, Venere, and Salerno (2000) reported that UVC at doses of 0.50 and 1.00 kJ m^−2^ significantly reduced the natural infection of Botrytis storage rot on strawberry fruits.

Although all the doses showed a prominent effect in controlling crown rot of Berangan banana fruit, the undesirable changes of browning discoloration on the peel surface ultimately had limited the application of UVC doses onto this commodity. Wade, Tan, and Kavanagh (1993) also observed the similar effect of UVC on peel browning on Williams Cavendish banana. The occurrence of peel browning by UVC irradiation could lower the quality of banana fruit and causing the fruits not able to be sold in market even though the disease was successfully being controlled. In this study, the dose of 0.01 kJ m^−2^ UVC was found to be the optimum dose that Berangan banana fruit could tolerate, with the lowest percentage of browning discoloration, that is, <20% on the surface of the fruit, whereas, the UVC doses from 0.02 to 0.05 kJ m^−2^ caused an increasing rate of peel browning on Berangan banana fruit.

Although UVC caused severe browning on fruit surface, the pulp tissue was found unaffected. The melanin‐like pigment observed on the peel surface was claimed of having similar properties as 3‐desoxyanthocyanidin compound (Freytag, 1933). This compound resulted from oxidative browning reaction of phenolic compound by polyphenol oxidase (PPO) enzymes (Ding & Ling, 2014). Besides banana, the prolonged exposure of UVC also caused browning on other fresh produces, such as tomato (Maharaj, Arul, & Nadeau, 1999), strawberries (Marquenie et al., 2002), and peaches (Stevens et al., 1998). In contrast, the peel color of persimmon (Khademi, Zamani, Ahmadi, & Kalantari, 2013) and Satsuma mandarin fruits (Shen et al., 2013) were not affected by the application of UVC treatment at 1.5 and 3.0 kJ m^−2^ doses, respectively.

Crown tissue is the main entrance for the pathogen invasion and the rot that is usually confined in the tissue would later penetrate into the pedicels and fingers of banana fruit (Wallbridge, 1981). The modifications of cell wall structure in the crown tissue of Berangan banana fruit were examined through scanning electron microscopy (SEM). The SEM micrographs showed that the dose of 0.01 kJ m^−2^ UVC had maintained the integrity of tissue structure until day 5, indicating the beneficial effect of low UVC dose in reducing the crown tissue damage, whereas, the control crown tissue showed rapid cell wall degradation due to an intense colonization by fungal mycelia (Figure 3). The damaged epidermal surface would cause water to be massively transpired from the tissue (Valero & Serrano, 2010). Charles, Goulet, and Arul (2008) observed that 3.7 kJ m^−2^ UVC successfully induced the decay resistance through cellular reinforcement on the epicuticular surface of tomato fruit. The loss of barrier strength in cell wall against pathogen penetration thereby increased the decay development.

### 4.2 Effect of UVC irradiation on physico‐chemical and antioxidant capacity of Berangan banana fruit during ripening

The effects of UVC irradiation (0.01 to 0.05 kJ m^−2^) on respiration rate, ethylene production, and physico‐chemical characteristics of Berangan banana fruit were evaluated. It was observed that fruit treated with 0.03 kJ m^−2^ UVC exhibited highest respiration rate occurred in the irradiated tissue. This finding was in agreement with the earlier reports on *Cucurbita pepo* where UVC raised the respiration rate in the fruit during storage (Erkan, Wang, & Krizek, 2001). Escalona, Aguayo, Martinez‐Hernandez, and Artes (2010) who studied the effect of UVC irradiation on postharvest quality of baby spinach leaves found that the samples treated with UVC from 7.2 to 24.9 kJ m^−2^ doses showed a higher respiration rate than control. The authors suggested that the effect was due to the significant tissue damage caused by UVC application that leads to the occurrence of respiratory stress.

On the other hand, it was found that UVC irradiation at 0.01–0.05 kJ m^−2^ tremendously reduced ethylene production of Berangan banana fruits compared to control fruits. This result was in agreement with Maharaj et al. (1999) who reported that UVC at 3.7 × 10^3^ and 24.4 × 10^3^ J m^−2^ were beneficial in suppressing the ethylene production of tomato fruits to the lower levels, that is, 1.2 and 1.1 μl kg^−1 ^hr^−1^, respectively, than control, that is, 1.6 μl kg^−1 ^hr^−1^ after 24 days of storage period.

Fruit firmness is one of the major quality indices that determine the fruit quality. An excessive fruit softening corresponded to deterioration of banana fruits and this would indicate that the fruits have a very short postharvest life. Generally, peel and pulp firmness of Berangan banana fruit decreased throughout the ripening days at holding temperature of 25 ± 2°C. This is mainly due to water losses and deterioration of cell wall structure during the ripening process (Tee, Ding, & Rahman, 2012). The breakdown of cell wall compositions such as pectin and cellulose by cell wall‐degrading enzymes and movement of water from the fruit peel to pulp are also contributing to the softening of banana fruit (Kader & Yahia, 2011).

Dose of 0.03 and 0.05 kJ m^−2^ UVC irradiation significantly retained peel and pulp firmness of banana at 3 DAR. However, the effect disappeared by 5 DAR. This result indicated that UVC irradiation could only delay the fruit softening at the turning yellow fruit (at 3 DAR), but this effect was transient as the fruit fully ripened at 5 DAR. This observation might explain that the ripening and senescence processes of banana fruit had caused the rapid changes in fruit firmness. Besides firmness, it was observed that the application of UVC at different doses did not affect the rate of weight loss of Berangan banana during ripening process. A similar observation was reported by Khademi et al. (2013) who found that UVC at 1.5 and 3.0 kJ m^−2^ doses did not significantly affect the changes of firmness and weight losses in persimmon fruit during storage, but the reduction was only due to the progression of storage period. Perkins‐Veazie, Collins, and Howard (2008) observed that UVC at 4 kJ m^‐2^ dose on blueberry fruit (*Vaccinium corymbosum* L.) did not affect the fruit firmness when compared to control during cold storage at 5°C for 7 days and followed by 2 days at 20°C.

Peel color is one of the crucial criterions that influence consumer purchase. It is also the primary indicator of fruit ripeness, eating quality, and shelf life (Thompson, 2003). All Berangan banana fruits experienced a color change from green to yellow during ripening, indicating that the fruits could undergo normal ripening after irradiated with UVC. The changes in peel color proved that UVC did not alter the event of chlorophyll breakdown in the peel of banana during ripening. However, it was observed that the rapid changes in peel lightness, chroma, and hue angle upon the application of UVC at the highest dose tested in this study was 0.05 kJ m^−2^. This was considered as an unfavorable effect of UVC on banana fruit which influenced a faster ripening process when applied at high doses.

Titratable acidity (TA) is also an important quality indicator for fruit shelf life. The organic acids contained in banana fruits are citric, malic, succinic, and oxalic acids (Tee et al., 2012). The acids are normally increased in banana during ripening. A faster increase in TA indicated that rapid senescence process has occurred in the fruit. In this study, the percentage of malic acid in Berangan banana fruit gradually increased in all treatments during ripening. This indicated that UVC‐treated fruits had similar ripening rate as control fruit. Exposure fruit to different UVC doses had no effect on its TA after 5 days of ripening, even though a slight decrease was observed in fruit treated with 0.02–0.05 kJ m^−2^ UVC doses when fruits were at 3 DAR. This suggested that the Berangan banana fruit TA was not affected by application of UVC irradiation as the changes were transient and comparable with controls as DAR progressed. This finding was similar to the effect of UVC on strawberry cv. Seascape, where the dose of 4.1 kJ m^−2^ did not affect TA of the fruit (Pan, Vicente, Martinez, Chaves, & Civello, 2004). In another study conducted by Gonzalez‐Aguilar, Wang, Buta, and Krizek (2001), UVC irradiation of 4.9 and 9.9 kJ m^−2^ did not alter the level of organic acids in mango fruits.

The highest UVC dose used in this study, that is, 0.05 kJ m^−2^ significantly increased the soluble solids concentration (SSC) in Berangan banana fruit. The similar dose also caused the reduction of pH in the fruit compared to the other treatments. In contrast, the lower doses applied, that is, 0.01–0.04 kJ m^−2^ UVC had retained the lower level of fruit SSC by the end of ripening, which reflects a delay in banana ripening and senescence processes. Similarly, the lower UVC irradiation doses exhibited a significant higher pH value than control, indicating UVC‐irradiated fruit was less acidic and had a slower rate of ripening process than controls. The faster increase in SSC and the higher percentage of pH decrease in 0.05 kJ m^−2^ UVC‐irradiated fruit indicated that the UVC caused acceleration in fruit ripening process. This is supported by Liu et al. (1993), who observed that the prolonged exposure of UVC had increased the ripening and senescence process in tomato fruits.

The ripening acceleration in fruit irradiated by 0.05 kJ m^−2^ UVC suggested that UVC might induce the synthesis of polysaccharides and organic acid compounds in fruit when applied at higher doses. The acceleration of ripening and senescence could also be related to the photo‐oxidation reactions promoted by UVC irradiation through production of reactive oxygen species (Shama & Alderson, 2005). The free radicals produced would modify the cell biochemical event in plant tissue including the enzyme activity, nucleic acids, cell membranes, and cell walls integrity which eventually leads to rapid senescence (Foyer, Descourvieres, & Kunert, 1994).

Total phenolic content is the main precursor to induce the antioxidant capacity in the plant tissues (Wang, Chen, & Wang, 2009). In this study, total phenolic content of Berangan banana fruit irradiated with 0.05 kJ m^−2^ UVC increased as DAR progressed. The results suggested that UVC irradiation at high dose probably acted as physical stress to induce the accumulation of phenolic compound. Liu, Cai, Lu, Han, and Ying (2012) also found that tomato cv. “Zhenfen 202” irradiated with UVC at doses of 2.0 to 16.0 kJ m^−2^ showed significant increments in phenolic content during storage at 14°C. Similarly, results from previous studies on tomato (Pataro, Sinik, Capitoli, Donsì, & Ferrari, 2015), strawberry (Erkan, Wang, & Wang, 2008), and blueberry fruit (Perkins‐Veazie et al., 2008) showed that UVC irradiation led to an increase in total phenolic compared to control fruit. The stimulation of phenolic biosynthesis in UVC‐treated bananas might explain the additional quenching activity on active oxygen species and thus improved health‐promoting effects in the fruits. Meanwhile, the low amount of total phenolic content or a sharp decrease in 0.03 kJ m^−2^ UVC‐treated fruits by 5 DAR might be due to the higher rate of respiration (Figure 4a), which resulted in the loss of phenolic content due to the degradation of certain phenolic compounds (Day, 2001).

UVC at dose of 0.03 kJ m^−2^ caused a significant increase in antioxidant capacity of Berangan banana fruit during ripening. The increase in antioxidants level could be related to the induction of defense mechanisms in fruit to react with oxidative stresses induced by UVC irradiation. This is supported by Wang et al. (2009), who reported that the blueberries cv. Duke irradiated with 2.15, 4.30, and 6.45 kJ m^−2^ UVC doses showed higher antioxidant capacity. The application of 1 to 4 kJ m^−2^ UVC doses also increased FRAP values in both cultivars of blueberries, that is, Collins and Bluecrop (Perkins‐Veazie et al., 2008).

## 5 Conclusions

In conclusion, the applications of UVC irradiation has reduced postharvest crown rot disease and maintain the quality attributes of Berangan banana fruit during ripening at 25 ± 2°C. UVC irradiation at 0.01 kJ m^−2^ could be considered as the optimum dose for Berangan banana, which effectively reduced disease severity without causing browning on the fruit peel. Moreover, UVC did not give adverse effect on respiration rate, ethylene production, weight loss, firmness, color changes, soluble solids concentration, titratable acidity, pH, and total phenolic content when applied at 0.01 kJ m^−2^ dose. However, the application of 0.05 kJ m^−2^ UVC induced the ripening by promoting rapid changes in color, SSC, and pH of the fruit. The application of UVC irradiation also found beneficial in improving health‐promoting values in banana fruits due to elevation of antioxidant levels. Less modification on epidermal surface and cell wall structures were observed in crown tissue when irradiated with 0.01 kJ m^−2^ UVC dose as compared to control. This study suggests that the application of UVC irradiation at 0.01 kJ m^−2^ dose might be a promising method in reducing the usage of postharvest fungicide.

## Conflict of Interest

None declared.
